# Garlic Derived Diallyl Trisulfide in Experimental Metabolic Syndrome: Metabolic Effects and Cardioprotective Role

**DOI:** 10.3390/ijms21239100

**Published:** 2020-11-30

**Authors:** Jovana N. Jeremic, Vladimir Lj. Jakovljevic, Vladimir I. Zivkovic, Ivan M. Srejovic, Jovana V. Bradic, Isidora M. Milosavljevic, Slobodanka Lj. Mitrovic, Nemanja U. Jovicic, Sergey B. Bolevich, Andrey A. Svistunov, Suresh C. Tyagi, Nevena S. Jeremic

**Affiliations:** 1 Department of Pharmacy, Faculty of Medical Sciences, University of Kragujevac, Svetozara Markovica 69, 34 000 Kragujevac, Serbia; jovana.jeremic@medf.kg.ac.rs (J.N.J.); jovanabradickg@gmail.com (J.V.B.); isidora.stojic@medf.kg.ac.rs (I.M.M.); 2 Department of Physiology, Faculty of Medical Sciences, University of Kragujevac, Svetozara Markovica 69, 34 000 Kragujevac, Serbia; drvladakgbg@yahoo.com (V.L.J.); vladimirziv@gmail.com (V.I.Z.); ivan_srejovic@hotmail.com (I.M.S.); 3 Department of Human Pathology, I.M. Sechenov First Moscow State Medical University, Trubetskaya Street 8, 119991 Moscow, Russia; bolevich2011@yandex.ru; 4 Department of Pathology, Faculty of Medical Sciences, University of Kragujevac, Svetozara Markovica 69, 34 000 Kragujevac, Serbia; smitrovic@medf.kg.ac.rs; 5 Department of Histology and Embryology, Faculty of Medical Sciences, University of Kragujevac, Svetozara Markovica 69, 34 000 Kragujevac, Serbia; nemanjajovicic.kg@gmail.com; 6 Research Institute of Pharmacy, I.M. Sechenov First Moscow State Medical University, Trubetskaya Street 8-2, 119991 Moscow, Russia; svistunov@sechenov.ru; 7 Department of Physiology, School of Medicine, University of Louisville, 500 S Preston Street, Louisville, KY 40202, USA; suresh.tyagi@louisville.edu

**Keywords:** diallyl trisulfide, garlic, metabolic syndrome, isolated rat heart

## Abstract

This study aimed to examine the effects of diallyl trisulfide (DATS), the most potent polysulfide derived from garlic, on metabolic syndrome and myocardial function in rats with metabolic syndrome (MetS). For that purpose, we used 36 male *Wistar albino* rats divided into control rats, rats with MetS and MetS rats treated with 40 mg/kg of DATS every second day for 3 weeks. In the first part, we studied the impact of DATS on MetS control and found that DATS significantly raised H_2_S, decreased homocysteine and glucose levels and enhanced lipid and antioxidative, while reducing prooxidative parameters. Additionally, this polysulfide improved cardiac function. In the second part, we investigated the impact of DATS on ex vivo induced ischemia/reperfusion (I/R) heart injury and found that DATS consumption significantly improved cardiodynamic parameters and prevented oxidative and histo-architectural variation in the heart. In addition, DATS significantly increased relative gene expression of eNOS, SOD-1 and -2, Bcl-2 and decreased relative gene expression of NF-κB, IL-17A, Bax, and caspases-3 and -9. Taken together, the data show that DATS can effectively mitigate MetS and have protective effects against ex vivo induced myocardial I/R injury in MetS rat.

## 1. Introduction

Cardiovascular diseases (CVDs) are a leading cause of premature death and a major chronic disability worldwide [1]. One of the serious CVDs, a growing health problem, is myocardial infarction (MI), which is commonly followed by extended ischemia and heart failure. Ischemia causes cardiac dysfunction, arrhythmias, myocardial infarction and sudden death, mediated by low pH values, low oxygen levels and disruption of K^+^ and Ca^2+^ concentrations [1]. Strategy in salvaging damaged myocardium in ischemia involves restoration of blood flow. However, the most common, activation of the complex inflammatory response in the reperfusion period leads to irreversible damage called reperfusion injury [2]. Therefore, ischemia/reperfusion (I/R) injury is typically occurring as a result of vessel occlusion followed by a set of stresses during the recovery of blood flow. The factors contributing to I/R injury are very complex and besides already mentioned include microvascular dysfunction, the release of reactive oxygen species (ROS), and activation of mitochondrial apoptosis and necrosis [3]. However, these mediators are common in the pathology of metabolic syndrome (MetS) as well. Namely, modified metabolism, damaged microvasculature and weakened immune system are the major MetS-related adverse effects on the cardiac system [4,5]. The number of MetS patients with ischemic heart disease is alarmingly increasing and long-term prognosis, as well as control of complications, remain poor. Therefore, it is necessary to improve cardioprotective strategies and find new agents against I/R injury (so-called preconditioning agents) to preserve myocardial capacity and reduce the risk of cardiovascular events in patients with MetS [3,4].

Garlic (*Allium sativum* L.) is a traditional food recognized as beneficial for numerous diseases since ancient times. Although the precise protective mechanisms are still unclear and require further explanation, many authors hypothesize that *Allium* species caused the most beneficial effects by sulfur compounds [6]. It is a well-known fact that abundant organosulfur compounds found in the garlic interact with thiol groups or thiol-containing compounds from biological systems and augments bioavailability of hydrogen sulfide (H_2_S) [6,7]. Additionally, numerous studies have found that as a gasotransmitter, H_2_S is key modulators of I/R injury and could alleviate heart damage probably by activating of adenosine triphosphate-sensitive potassium channels (K-ATP) [7], affecting several pro-inflammatory cytokines [8] and decreasing hydrogen peroxide in parallel with increasing GSH levels [9]. Raw intact garlic bulbs contain high amounts of γ-glutamylcysteine, which can undergo hydrolysis or oxidation and at the end decompose to sulfur-containing compounds. The most important organosulfur compound isolated from garlic is diallyl trisulfide (DATS) also known as allitridin or 4,5,6-trithia-1,8-nonadiene. DATS, as one of the most stable and safe naturally occurring polysulfide, is an attractive H_2_S donor for in vivo studies with an eye to clinical relevance [10,11].

Findings from our previous study referring to blood-glucose-lowering and cardioprotective effects of DATS in chemically induced type 1 diabetes mellitus [12] encouraged us to examine the effect of DATS on the more complex disease, which involve a group of pathophysiological abnormalities, such as MetS. To our best knowledge, there are no available reports that explain the role of DATS consumption in MetS, which provide the precise association between blood pressure, glycemia and lipid profile, as well functional and morphological properties of the heart after DATS administration. Therefore, the present study aimed to examine the effects of DATS on MetS and its potential cardioprotective role in ex vivo-induced cardiac I/R injury in rats with MetS.

## 2. Results

### 2.1. Anti-Metabolic Syndrome Effects of DATS

#### 2.1.1. Biochemical Parameters

**Figure 1:**
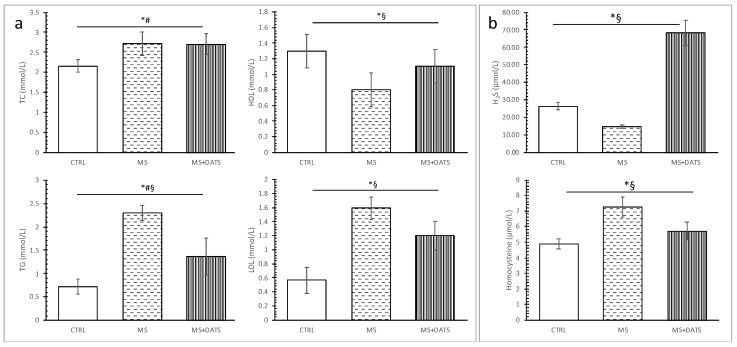
Effects of diallyl trisulfide (DATS) consumption on (**a**) lipid parameters: TC—total cholesterol, TG—triglycerides, HDL—high-density lipoprotein and LDL—low-density lipoprotein; (**b**) H_2_S—hydrogen sulfide and homocysteine levels. Values are presented as mean ± SD, n = 12, per group. * *p* < 0.05 CTRL vs. MS, # *p* < 0.05 CTRL vs. MS+DATS, § *p* < 0.05 MS vs. MS+DATS.

All measured lipid parameters were significantly increased except high-density lipoprotein (HDL), which was decreased in rats with MetS compared to CTRL. DATS consumption in MetS rats significantly reduced triglycerides (TG) and low-density lipoprotein (LDL) and significantly increased HDL levels (Figure 1a).

Three weeks of DATS consumption resulted in a statistically significant increase of serum H_2_S levels in rats with metabolic syndrome compared to untreated rats. In contrast to these results, homocysteine levels were significantly higher in the MS group, compared with the CTRL and MS+DATS groups (Figure 1b).

#### 2.1.2. Insulin and Glucose Levels during Oral Glucose Tolerance Test (OGTT)

**Figure 2:**
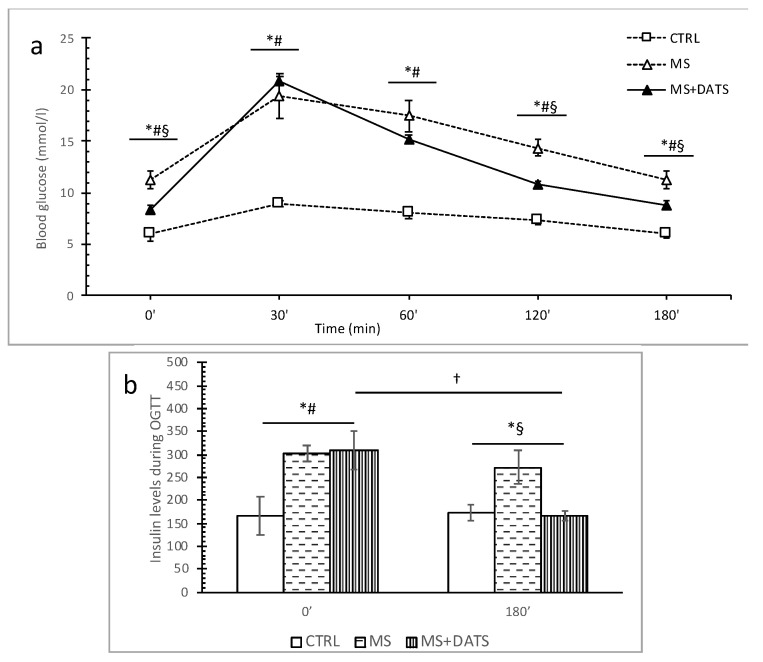
Effect of DATS consumption on glucose and insulin level during oral glucose tolerance test (OGTT). (**a**) Fasting blood glucose level (0′) and blood glucose levels 30′, 60′, 120′ and 180′ after an oral glucose loading (2 g/kg body weight); (**b**) fasting insulin levels and 180’ after an oral glucose loading. Values are presented as mean ± SD, n = 6, per group. * *p* < 0.05 CTRL vs. MS, # *p* < 0.05 CTRL vs. MS+DATS, § *p* < 0.05 MS vs. MS+DATS, † *p* < 0.05 MS at 0′ vs. MS at 180′.

Fasting blood glucose levels were significantly increased in both groups of MetS rats (MS and MS+DATS) compared to the CTRL group. Furthermore, DATS consumption significantly decreased glucose level (fasting blood glucose, as well as 120 and 180 min after an oral glucose loading) (Figure 2a).

Similar, fasting insulin concentrations were significantly increased in MetS rats (MS and MS+DATS) compared with CTRL; meanwhile, 180 min after an oral glucose loading, insulin level was significantly increased in MS both when compared to CTRL and MS+DATS groups (Figure 2b).

#### 2.1.3. Redox Status

**Figure 3:**
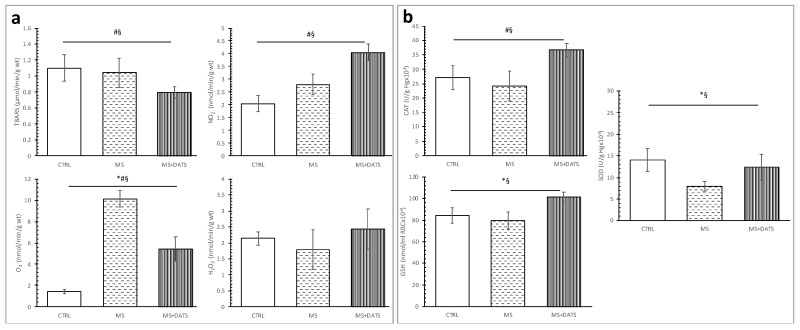
Effects of DATS consumption on oxidative stress parameters; (**a**) pro-oxidants: TBARS—index of lipid peroxidation, O_2_^−^—superoxide anion radical, NO_2_^−^—nitrite, H_2_O_2_—hydrogen peroxide; (**b**) antioxidants: CAT—catalase, GSH—reduced glutathione peroxide and SOD—superoxide dismutase. Values are presented as mean ± SD, n = 12, per group. * *p* < 0.05 CTRL vs. MS, # *p* < 0.05 CTRL vs. MS+DATS, § *p* < 0.05 MS vs. MS+DATS.

TBARS was significantly decreased, while nitrites (NO_2_^−^) were significantly increased in DATS treated rats with MetS compared with untreated rats (CTRL and MS groups). Besides, the superoxide anion radical (O_2_^−^) level was significantly increased in the MS group in comparison with CTRL and MS+DATS (Figure 3a).

On the other hand, superoxide dismutase (SOD) and reduced glutathione (GSH) were significantly decreased in MS group compared to CTRL. Importantly, treatment with DATS resulted in a significant enhancement of catalase (CAT), SOD activity and increased GSH amount (Figure 3b).

#### 2.1.4. In Vivo Cardiac Function

**Figure 4:**
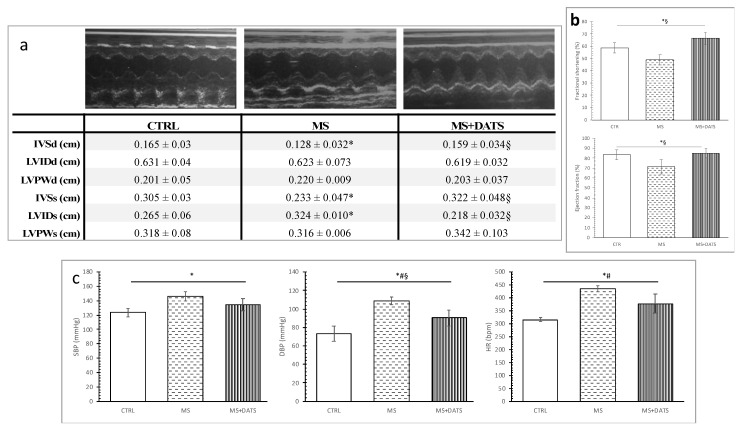
Effects of DATS consumption on in vivo cardiac function: (**a**) cardiodynamic parameters, representative M-mode echocardiography findings; (**b**) fractional shortening and ejection fraction; (**c**) SBP—systolic blood pressure, DBP—diastolic blood pressure and HR—heart rate. Values are presented as mean ± SD, n = 12, per group. * *p* < 0.05 CTRL vs. MS, # *p* < 0.05 CTRL vs. MS+DATS, § *p* < 0.05 MS vs. MS+DATS.

MetS rats had significantly changed echocardiographic parameters (decreased diastolic and systolic interventricular septal wall thickness (IVS), as well as increased left ventricular internal diameter (LVIDd)), but DATS treatment prevented these alterations (Figure 4a). Similar, percentages of fractional shortening and ejection fraction were decreased in rats with MetS, while DATS consumption significantly improved these parameters (Figure 4b).

Blood pressure and heart rate were significantly increased in rats with MetS compared with CTRL, while 3 weeks of DATS consumption significantly decreased only diastolic blood pressure (Figure 4c).

### 2.2. Efficiency of DATS Preconditioning in Rats with MetS

#### 2.2.1. Cardiodynamic and Oxidative Stress Parameters

**Figure 5:**
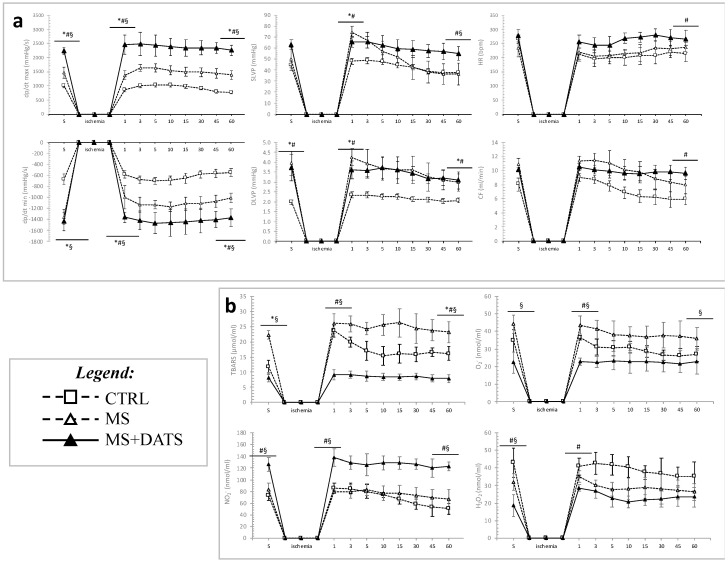
Preconditioning effects of DATS on (**a**) cardiodynamic parameters: dp/dt max—the maximum rate of LV pressure development, dp/dt max—minimum rate of LV pressure development, SLVP—systolic LV pressure, DLVP—diastolic LV pressure, HR—heart rate and CF—coronary flow; (**b**) pro-oxidants: TBARS—index of lipid peroxidation, O_2_^−^—superoxide anion radical, NO_2_^−^—nitrite, H_2_O_2_—hydrogen peroxide. Values are presented as mean ± SD, n = 12, per group. * *p* < 0.05 CTRL vs. MS, # *p* < 0.05 CTRL vs. MS+DATS, § *p* < 0.05 MS vs. MS+DATS.

Parameters of left ventricular contractile force (dp/dt max and dp/dt min, at all points of interest) and parameters of left ventricular pressure (SLVP and DLVP) were significantly altered as follows MS+DATS > CTRL > MS. Moreover, heart rate (HR) was higher in MS+DATS then in the CTRL group at the last minute of reperfusion (Figure 5a).

Furthermore, MetS rats had significantly increased values of the index of lipid peroxidation (measured as TBARS) and O_2_^−^ in coronary venous effluent, while DATS treatment significantly decreased levels of TBARS, O_2_^−^ and hydrogen peroxide (H_2_O_2_), an increased level of NO_2_^−^ (Figure 5b).

#### 2.2.2. Pathohistological Evaluation of Myocardial Tissue

**Figure 6:**
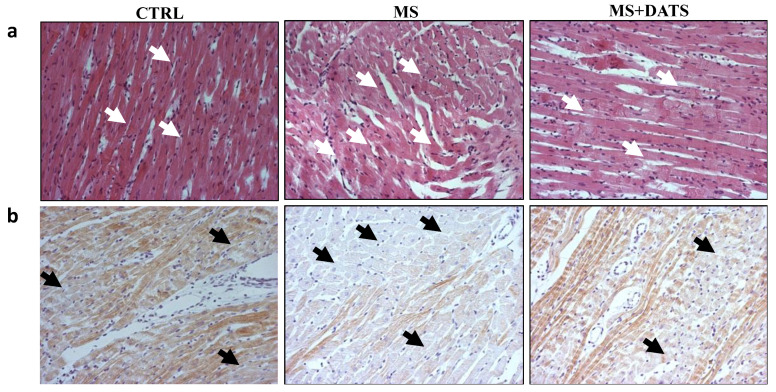
Representative heart tissue sections of (**a**) hematoxylin/eosin-staining (white arrows indicate representative changes in the heart tissue); (**b**) cTnT-immunostaining (black arrows indicate the area with the least colored cTnT in the heart tissue). Original magnification 20×.

I/R-induced cardiac histo-architectural variation was analyzed using hematoxylin/eosin staining that confirms the perturbed structure in MetS rat heart. Namely, in MS group extensive edema, cell swelling, and tissue necrosis (infarcted) zone with separation of muscle fibers were found. On the other hand, histological alterations were considerably reduced in DATS-treated group where mild edema, reduced inflammatory process and reduced necrotic area were obtained (Figure 6a). These ultrastructural changes were further confirmed with cardiac troponin T (cTnT) staining. As shown in Figure 6b in MS group there was an obvious loss of cTnT staining and slightly positive (brown) staining, while in the MS+DATS group, there was a decrease in staining with more intense (brown color) remaining found.

**Figure 7:**
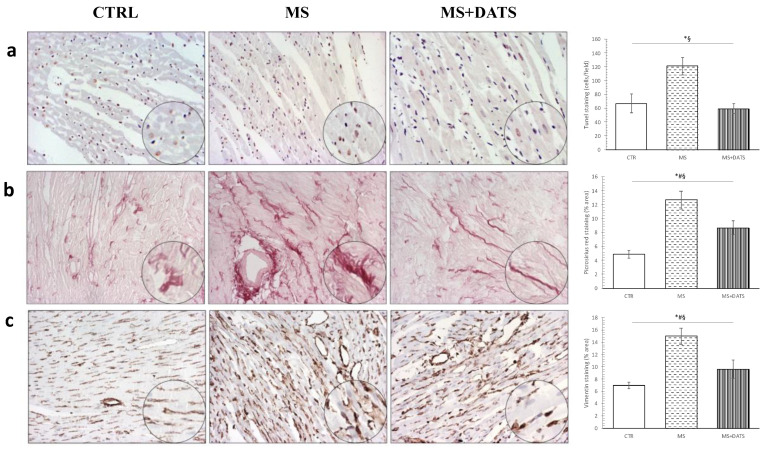
Representative heart tissue sections of (**a**) immunostaining of myocytes with terminal deoxynucleotidyl transferase dUTP nick end labeling (TUNEL) positive nucleus; (**b**) picrosirius red staining; (**c**) vimentin content. Values are presented as mean ± SD. * *p* < 0.05 CTRL vs. MS, # *p* < 0.05 CTRL vs. MS+DATS, § *p* < 0.05 MS vs. MS+DATS. Original magnification 20× and 40×.

More importantly, DATS treatment decreased the incidence of cardiomyocytes with and terminal deoxynucleotidyl transferase dUTP nick end labeling (TUNEL) positive nucleus, DNA fragment and apoptosis activity compared to untreated rats (MS+DATS vs. MS) (Figure 7a). Additionally, analysis of Picrosirius red and vimentin content using ImageJ revealed a significant increase in the amount of fibrosis in rats with MetS compared to the CTRL group. On the other hand, DATS consumption significantly decreased fibrosis (MS+DATS vs. MS) (Figure 7b,c).

**Figure 8:**
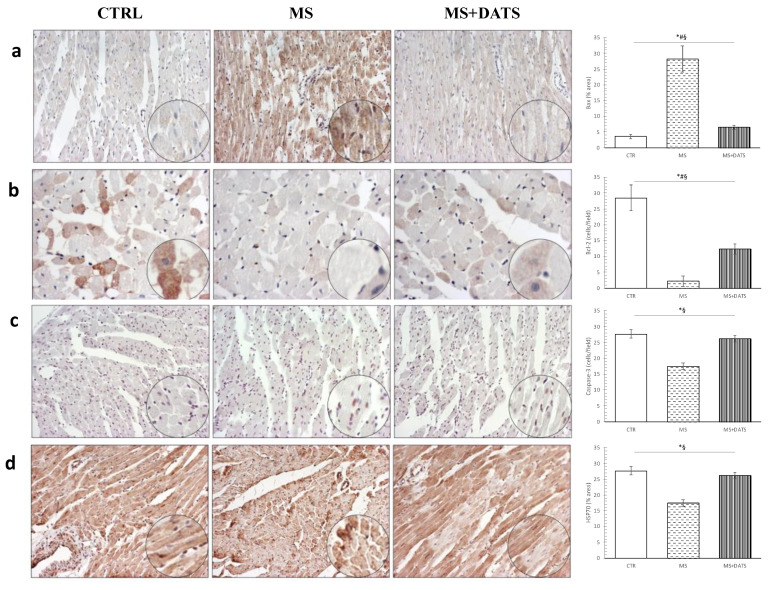
Representative heart tissue sections of immunohistochemical staining of (**a**) Bax; (**b**) Bcl-2; (**c**) Caspase-3; (**d**) heat shock protein 70. Values are presented as mean ± SD. * *p* < 0.05 CTRL vs. MS, # *p* < 0.05 CTRL vs. MS+DATS, § *p* < 0.05 MS vs. MS+DATS. Original magnification 20× and 40×.

Immunohistochemical analysis showed that in rats with MetS, Bax and Caspase-3 protein expression were significantly increased, while Bcl2 and HSP70 protein expression were significantly decreased in myocardium when compared to CTRL rats. By the DATS consumption, alteration of the expression of all mentioned protein was prevented and more similar to CTRL than to MS group (Figure 8a–d).

**Figure 9:**
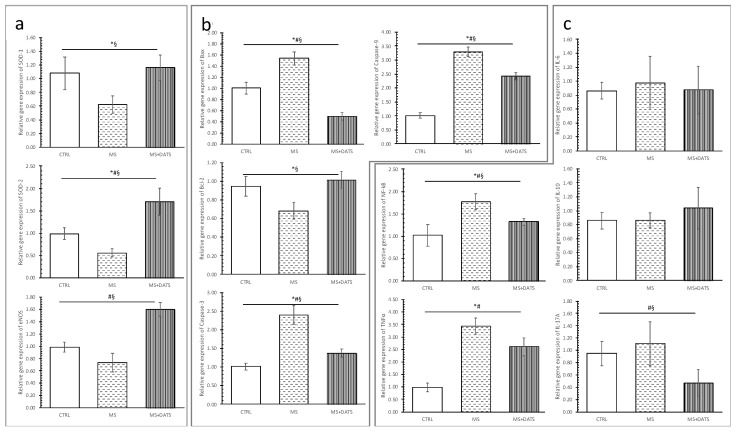
Effects of DATS consumption on relative gene expression of: (**a**) antioxidative: SOD-1—superoxide dismutase 1, SOD-2—superoxide dismutase 2, eNOS—endothelial nitric oxide synthase; (**b**) (anti)apoptotic: Bcl-2—B-cell lymphoma 2, Bax, caspase-3, caspase-9; (**c**) (anti)inflammative parameters: NF-κB, TNF-α, IL-6, IL-10, IL-17A. Values are presented as mean ± SD, n = 12, per group. * *p* < 0.05 CTRL vs. MS, # *p* < 0.05 CTRL vs. MS+DATS, § *p* < 0.05 MS vs. MS+DATS.

Relative gene expression of endothelial nitric oxide synthetase (eNOS), SOD-1 and SOD-2 were significantly decreased in MS group compared to CTRL, while DATS consumption significantly increased relative gene expression of these parameters (Figure 9a).

On the other hand, relative gene expression of Bax, caspase-3 and caspase-9 were significantly increased, while Bcl-2 was significantly decreased in MS compared to the CTRL group. Contrarily, DATS consumption significantly decreased relative gene expression of Bax and both determined caspases and increased relative gene expression of Bcl-2 (Figure 9b).

Relative gene expression of NF-κB and TNF-α were significantly increased in MS compared to CTRL, while DATS treatment significantly decreased the relative gene expression of NF-κB and IL-17A (Figure 9c).

## 3. Discussion

Although DATS as a H_2_S donor has been described several decades ago, many aspects of its effects on the cardiovascular system and metabolic disorders remain poorly known. In the present study, it was demonstrated for the first time that DATS can effectively mitigate MetS and have protective effects against ex vivo induced myocardial I/R injury in MetS rat.

MetS as a heterogeneous disorder is characterized by insulin resistance, pancreatic beta-cell dysfunction, hypertension, obese and hyperlipidemia [13]. To stimulate MetS in rats we used an animal model which involves a high-fat diet (HFd) for 4 weeks, followed by single, low-dose of streptozotocin (STZ) injection. Based on literature data, these two stressors in combination are great for experimental model because they mimic the pathology of MetS and T2DM in humans [14,15]. Besides, consumption of HFd is prevalent all over the world, which also supports the fact that used model is suitable because it is comparable to MetS in humans [14]. 

In the first part of our study, we were focused on the impact of DATS on metabolic control of disease, such as lipidemic and glycemic profile. In our experimental model, MetS rats displayed elevated levels of glucose, TC, TG, LDL and declined levels of HDL compared to healthy rats which are in correlation with manifestations of MetS described in the literature. On the other hand, 3 weeks of DATS consumption had a strong influence on metabolic parameters, which was manifested in a decrease of TG and LDL as well increase of HDL (Figure 1a). These results agree with those described by other authors who have examined different garlic-based extracts and reported an improved lipid profile [16,17]. The possible protective effects of garlic and DATS may be due to indirect or direct inhibition of endogenous cholesterol synthesis and/or due to changes in the portions of lipoprotein cholesterol fractions [18]. Previous studies indicate that ingestion of garlic is linked with inhibition of hepatic fatty acid synthesis mainly by lowering activities of the crucial enzymes and consequently by reducing lipid accumulation in the liver and TG in the plasma [19]. This agrees with other research which showed that supplementation with garlic extracts significantly inhibit the increment of lipid levels in serum due to inhibition of hydroxyl methyl glutaryl coenzyme A (HMG-CoA) reductase, which plays one of crucial role in controlling lipid levels in plasma and various tissues [20]. A recent study shows that NaHS (inorganic H_2_S donor) extremely reduces TG serum levels in mice fed with HFd through stimulation of 5’ adenosine monophosphate protein kinase (AMPK) and subsequent autophagic responses. NaHS by increasing H_2_S activates the flux of autophagy in the liver, AMPK blocks mTOR which in turn activates the autophagy pathway [21]. DATS as an H_2_S donor probably may influence TG level via the same pathways. The garlic consumption in MetS also leads to a decrease in glucose levels with reduced insulin resistance and increased insulin sensitivity [22], which was obtained as well in our study after the introduction of DATS (Figure 2). Furthermore, several papers reported the hypoglycemic activity of garlic is attributed to sulfur compounds, such as DATS and others: diallyl disulfide (DADS), diallyl sulfide (DAS), alliin, allicin and ajoene, due to their free -SH group [23,24]. Decreased blood glucose levels after garlic consumption might be explained by the diminution of glucose absorption from the gastrointestinal tract by garlic and its active compounds [25].

Increasing evidence in animal and human studies shows that oxidative stress plays a central role in the pathogenesis and complications of MetS. Free radicals generated by glucose oxidation in combination with the loss of endogenous antioxidants can lead to damage of organelles in the cells and the development of insulin resistance [26]. Since, in a previously published paper, we noticed that DATS could enhance antioxidative parameters in rats with chemically induced diabetes mellites type 1 [12], we hypothesized that DATS as a powerful antioxidant may also help in alleviating MetS and MetS-related complications. We obtained a significant reduction of prooxidants and increment of antioxidants (Figure 3), which is in agreement with the previous data describing the effects of whole garlic, garlic oil, extracts and its active compounds [23,27,28]. DATS consumption was effective in the reduction of TBARS and O_2_^−^ in plasma of MetS rats (Figure 3a), which was supported by an increase in the activities of all measured antioxidative parameters (Figure 3b). DATS treatment in our study was able to restore GSH levels which are known to be reduced in MetS due to deficiency in GSH precursor amino acids [29]. Furthermore, studies have shown that hyperglycemia can induce c-Jun N-terminal kinase (JNK) dependent nuclear factor (NF-κB) activation through the generation of ROS and cause cell apoptosis. The DATS treatment can suppress cardiomyocyte apoptosis by inhibiting JNK/NF-κB signaling via attenuating ROS generation [11].

Another aspect of our study was based on the determination of cardiac function as an important pathogenic feature of MetS. In some studies, Hcy is described as an independent risk factor, while in others it is defined as merely a risk marker. Regardless of the current controversies, Hcy and hyperhomocysteinemia (HHcy) are the new-multifactorial risk factors which interact with CVD [30]. Therefore, we attempted to discover the Hcy and HHcy relationship during the chronic consumption of DATS in rats with MetS. In our study, there was a significant increase in the level of Hcy which was expected since the rise of Hcy is linked to non-insulin-dependent diabetes mellitus. These results are in correlation with the findings referring to antioxidative stress markers since is been reported that auto-oxidation of Hcy contributes to the additional generation of ROS [31]. On the contrary, DATS intake was capable of reducing the Hcy levels thus exerting protective effects on the cardiovascular system (Figure 1b). Mechanisms by which DATS mediates beneficial effects on Hcy include modulation of variety intracellular signaling process but details remain unclear. It has been previously proved that elevated plasma levels of Hcy, are followed by a drop of endogenous production of H_2_S [31]. In our study, decreased Hcy supports an increase of H_2_S and might explain our results. By increasing the level of H_2_S, lipid hydroperoxides in the oxidized LDL are destroyed and the atherogenic potential of LDL decreases [32]. This also may be one of the possible explanations for the hypolipidemic effects of DATS consumption. Reducing homocysteine levels and increasing H_2_S in the serum directly reduces both the risk of cardiovascular disease and the extent of damage to other organs. Chronic administration of DATS significantly decreased DBP and HR in rats with MetS (MS+DATS vs. MS) (Figure 4c), which is consistent with the previous researchers. Blood pressure-lowering effects can be explained by H_2_S production [33] and a decrease in peripheral vascular resistance [34]. Moreover, data suggest that NO and H_2_S endothelium-dependent and K^+^-ATP-dependent vasodilatory effects [35,36]. In that sense, increased production of NO as well as H_2_S by DATS intake might be an explanation for obtained hypotensive effects. Furthermore, an echocardiographic examination, performed after chronic consumption of DATS on rats with MetS (MS+DATS), illustrated a significant increase of IVSd, IVSs, FS and decrease of LVIDs, compared to MS group (Figure 4a,b). Increase in FS and EF in combination with a decrease in systemic blood pressure (BP) manifests the improvement in systolic and contractile functions in rat heart from the MS+DATS group.

Since MetS is a significant risk factor for heart diseases and myocardial ischemia [37], in the second part of our study special focus was given to examine the effects of DATS on ex vivo induced I/R injury. We found markedly decreased values of SLVP, DLVP and CF during the reperfusion period in metabolic conditions. On the other hand, the values of these parameters in DATS-treated group remain almost constant over 30 min of reperfusion (Figure 4a,b), which thus indicated undisturbed heart function. Due to the accumulation of endogenous metabolites in the heart during I/R, there is a series of complex cellular and humoral reactions which induce arrhythmias, morphological changes in the myocardial tissue and harmful effects. Blockade of endogenous H_2_S synthesis may also induce the modulation of cellular electrophysiology and arrhythmias, especially at the level of ion channels [38]. Therefore, the absence of significant fluctuations in cardiodynamic parameters directly shows the beneficial effects of DATS consumption in rats with MetS. Mechanisms involved in cardioprotection probably include the opening of the K-ATP channels, activation of PKC [39] and ROS scavenging [40], which together reduce the Ca^2+^ influx and shorten the action potential [38]. Among them, cardioprotection provided by activation of K-ATP and PKC most probably plays a central role [41].

The underlying mechanisms of ex vivo I/R injured heart include many reactive processes, such as oxidative stress, inflammation and apoptosis. These biological reactions stimulate each other and cause anatomical and functional changes [38,39,40,41]. In our study, we confirmed that chronic DATS administration protects against the I/R injury in the heart. Specifically, the improvement of cardiodynamic parameters was accompanied by antioxidative, anti-inflammatory and anti-apoptotic effects. Our results have shown that DATS consumption inhibits ROS in effluent and increase relative gene expression of antioxidative parameters in LV of rat heart with MetS. As it is presented in Figure 5b, DATS consumption significantly decreased levels of TBARS, O_2_^−^ and H_2_O_2_ in the stabilization period and during the whole reperfusion period. These results were quite expected because DATS can induce the generation of H_2_S, in a thiol-dependent manner, which is well known as an activator of endogenous antioxidant defenses and inhibitor of pro-oxidants. Calvert and coworkers suggested that these effects are provided through the Nrf-2-dependent pathway [42]. In correlation with these, our results highlighted that DATS consumption increased the relative gene expression of SOD-1 and SOD-2 in LV (Figure 7a). Additionally, the increment of SOD directly affects the reduction of cardiomyopathy. These findings are in correlation with previous, which confirmed both exogenous and endogenous derived H_2_S exhibit powerful cytoprotective effects in different models of heart failure [11]. Consequently, excessive ROS production and decrement of antioxidative parameters through modulation of the heart structure and stimulation of numerous inflammatory and apoptotic markers can underline pathological processes associated with I/R injured MetS heart. Besides the already mentioned roles of DATS in ROS balancing, we additionally examined its effects on HSP70 (Figure 8d). This is of great interest since it is well known that free radicals especially the superoxide anion radical directly triggers an early increase of this protein in the I/R injured isolated rat heart. These findings suggest that although ROS may cause necrosis of myocytes at the same time may mediate HSP70, which prevents apoptosis and plays a role in the second window of protection [43,44]. On the other hand, in our study of 3 weeks, DATS consumption prevented necrosis in rats with MetS (Figure 6a), while HSP70-expression in heart tissue was slightly higher in this group of rats compared to the untreated group of rats (Figure 8d).

In our study, DATS consumption significantly reduced edema, inflammatory process and necrotic area (Figure 6) in heart tissues of MetS rats. Bearing in mind our results, DATS has the potential to preserve both function and structure of the heart under ischemic conditions. While examining the effects of DATS on (anti)inflammatory markers in LV, we found significantly reduced expression of NF-κB and IL-17A (Figure 9c). There is strong evidence supporting that garlic and garlic compounds inhibit the expression of cell adhesion molecules through downregulation of intracellular transduction pathways, such as AP-1 and JNK or NF-κB [11]. Preconditioning with NaHS preserved heart and kidney from degradation and translocation of NF-κB. In the literature data, there is great uncertainty whether H_2_S has pro-inflammatory or anti-inflammatory properties. However, based on our results, DATS as an H_2_S donor provides anti-inflammatory effects [7,45]. Inflammation with elevated immunocompetent cells in the cardiac tissue plays a pivotal role in the pathophysiology of cardiomyopathy and it is usually governed by several regulatory genes mediated by apoptosis signals. Therefore, we aimed to access the impact of DATS on (anti)apoptotic markers in LV of the heart. In the hearts of MetS rats subjected to I/R injury, relative gene expression of Bax, caspase-3 and caspase-9 were significantly increased, while Bcl-2 was significantly decreased compared to I/R group. On the other hand, chronic DATS consumption significantly decreased relative gene expression of Bax and both determined caspases, while the relative gene expression of Bcl-2 was increased (Figure 9b). TUNEL assay also confirmed the antiapoptotic effect of DATS (Figure 7a). We found that results of relative gene expression are in correlation with protein expression (Figure 8) and taken together, our results collectively demonstrate that DATS can protect the heart against ex vivo induced I/R injury in MetS hearts, probably by its ability to ameliorate oxidative stress, apoptosis and inflammation in the heart. Our results are in line with previous reports [46] suggesting antiapoptotic effects of DATS have been attributed to H_2_S and its possibility to phosphorylate few survival proteins, such as ERK, PI3K and Akt [42].

This study shows that the cardioprotective effects of DATS might be mediated through different cell responses. Although endothelial reticulum and oxidative stress are considered as two independent cellular pathways it is like to be, they are related. Stress responses protein such as HSP70 is required for mediating antioxidant mechanisms against Hcy induced cell damage. In this study, we showed that DATS mediate promotion of HSP70 and couple of pathways probably are responsible for overall cardioprotective effects of DATS. As a strong antioxidant DATS diminishes ROS production and together with an increment of HSP70 prevents cell damage leading to inhibition of caspase-3 activation, with consequent reducing of apoptosis [47].

## 4. Materials and Methods

### 4.1. Ethical Statement

Procedures used in this research were approved by the Ethics Committee for experimental animal well-being of the Faculty of Medical Sciences, University of Kragujevac (Kragujevac, Serbia), number: 01-1811 and were carried out following European Directive for the welfare of laboratory animals number: 2010/63/EU (22 September 2010) and principles of Good Laboratory Practice. Additionally, all experimental procedures have performed following the prescribed regulations of European Union Directive for the Protection of the Vertebrate Animals used for Experimental and other Scientific Purposes 86/609/EES and the principles of ethics.

### 4.2. Experimental Animals

*Wistar albino* rats used in this study have obtained from the Military Medical Academy, Belgrade, Serbia and housed under controlled regular environmental conditions: temperature (22 ± 2 °C), humidity and illumination (light/darkness cycle, 12/12 h). To adapt to the new environment, rats were caged seven days before the study began. Appropriate diets, standard (9% fat, 20% protein, 53% starch) or HFd (25% fat, 15% protein and 51% starch), and water have provided ad libitum.

All *Wistar albino* rats used in this study were male and were 13 weeks old at the time of sacrifice. At the beginning of the study 36 rats were randomly divided into healthy (CTRL, n = 12) and rats with MetS (n = 24). To induce MetS rats (6 weeks old, bodyweight 180 ± 20 g) were for four weeks fed with HFd and after 12-h starvation intraperitoneal single injection of STZ in the dose of 25 mg/kg was administrated. Three days (72 h) later, fasting blood glucose and insulin as well as blood pressure are measured. Animals with fasting blood glucose level over 7 mmol/L, fasting insulin level above 6 mmol/L and blood pressure level over 130/90 mmHg were included in the study and were considered as rats with MetS [48]. After successful MetS-induction rats were randomly divided into two subgroups: MS (untreated MetS rats) and MS+DATS (MetS rats treated with DATS) and both groups continued with HFd consumption.

### 4.3. DATS Treatment

To mimic the most common and the easiest administration route, DATS was applied by oral gavage (*per os*), every second day, at a dose of 40 mg/kg of body weight for three weeks [49].

DATS (purity ≥ 98%) was purchased from Sigma-Aldrich Chemie GmbH Eschenstrase 5, 82024 Taufkirchen, Germany. DATS was stored at −20 °C in dark, original bottles, according to the manufacturer’s recommendations.

### 4.4. Biochemical Parameters

The following lipid parameters: total cholesterol (TC), triglycerides (TG), high- and low-density lipoprotein (HDL and LDL) were analyzed spectrophotometrically on a programmed biochemical analyzer (Dimension Xpand, Siemens, IL, USA) in serum using commercial kits (Siemens Healthcare Diagnostics, Frimley, Camberley, Surrey, UK).

The methylene blue method was used for measuring total sulfide levels in the plasma. The supernatant was collected and total sulfide using a standard calibration NaHS curve was determined at a wavelength of 670 nm [50]. For determination, we used a microplate reader (Zenyth, Anthos, UK).

Homocysteine (Hcy) concentration was measured in serum with high-performance liquid chromatography (HPLC) procedure with reverse-phase separation and fluorescence detection, as described earlier. The fluorescence was measured at 390 nm, while emission was measured at 470 nm [51].

### 4.5. Oral Glucose Tolerance Test (OGTT), Insulin and Glucose Levels Determination

At the end of experimental treatment with DATS, OGTT was performed. After an overnight (12 h) fasting, the blood samples were collected from the tail-tip of the rats to determine the blood glucose and insulin levels (marked as 0). Next, glucose in a dose of 2 g/kg body weight was administered orally, and blood samples were taken by tail bleeding at 30, 60, 120 and 180 min after glucose loading. Glucose levels were immediately estimated at 0, 30, 60, 120 and 180 min, using the Accu-Chek (Roche Diagnostics, Indianapolis, IN, USA) glucometer with its comparing strips. Insulin levels were evaluated in plasma samples by the enzyme-linked immunosorbent assay (ELISA) method, as previously explained at 0 and 180 min [12,48].

### 4.6. Redox Status

Assessments of systemic pro-oxidant and antioxidant parameters were carried out in blood samples collected in the moment of animals sacrificing. Firstly, whole blood samples were centrifugated to separate the red blood cells (RBCs) from the plasma. In brief, during the centrifugation process, the RBCs are deposited at the bottom of the Vacutainer tubes (red layer), while plasma remains on the surface as an upper phase (yellow layer). After separation of the plasma, the RBCs were washed and stored in lysed forms.

In plasma samples, the levels of prooxidative parameters index of lipid peroxidation (measured as thiobarbituric acid reactive substances (TBARS)), nitrites (NO_2_^−^), superoxide anion radical (O_2_^−^), and hydrogen peroxide (H_2_O_2_) were measured, while in the lysate of erythrocytes, the activity of enzymatic defense system by evaluating of catalase (CAT) and superoxide dismutase (SOD), as well as amount of non-enzymatic antioxidant, reduced glutathione (GSH), were determined.

To determine lipid peroxidation in the plasma, we used an indirect method of determining the concentration of thiobarbituric acid reaction product (thiobarbituric acid reactive substances (TBARS)). The used method is based on the determination of lipid peroxidase level in the reaction of the malondialdehyde with thiobarbituric acid. Distilled water instead of plasma sample was used for the blind test.

To determine nitrite, we used an indirect method for determining the number of released nitrites. Actually, in 0.1 mL perchloric acid we pipetted 0.4 mL ethylenediaminetetraacetic acid (20 mM) and 0.2 mL of plasma samples. Then, we vortexed and incubated 15 min on ice and centrifugated 15 min (6000 rpm). Next, in sludge, we added 220 μL potassium carbonate. From there we took 220 μL samples and added 250 μL of freshly prepared Griess reagent and 125 μL of buffer for NO. After 15 min, we measured on 550 nm wavelength. Instead of plasma samples, the corresponding quantity of distilled water was used as a blind test.

The quantification of superoxide anion radical is based on the reaction of O_2_^−^ with nitro blue tetrazolium until nitro formazan blue. The determination was performed in plasma samples in the following steps: in 50 μL of plasma samples, we added extempore 950 μL of assay mixture and measured on 550 nm three times every 60 s. Instead of plasma samples, the comparing amount of distilled water was utilized as a blind test. Reaction responsible to H_2_O_2_ is based on the oxidation of phenol red by the reaction of hydrogen peroxide catalyzed by enzyme peroxidase from horseradish (HRPO).

To determine the hydrogen peroxide (H_2_O_2_) in 200 μL of plasma samples we added 800 μL of phenol red solution and 10 μL peroxidase (POD). And after incubation at room temperature for 10 min we measured the concentration of H_2_O_2_ at 610 nm. Instead of plasma, for a blind test, we used distilled water. Measurement must be in the time interval from 5 to 60 min because that is a period of generation and release of H_2_O_2._

In our study, the catalase was determined in lysate RBCs by dissolving lysate with distilled water in a 1:7 ratio, followed by the addition of ethanol in a 0.1:1 ratio (mixture). Then, in a 50 μL CAT buffer, we added 100 μL of the mixture and 1 mL of 10 mM H_2_O_2_ into the test tube. For the blind test, we used distilled water instead of sample. Every sample was measured six consecutive times at a wavelength of 360 nm. The final values were obtained based on a calculation scheme that involves subtraction and then the arithmetic means of the obtained values.

To determine SOD activity in 100 μL lysate RBCs we pipetted 1000 μL of carbonate buffer and then vortexed and added 100 μL of adrenaline. This method is based on the epinephrine method by Beutler. For the blind test, we used just mentioned solutions, and all samples, as well as a blind test, were measured at 470 nm wavelength. To measure the activity of GSH in the erythrocyte lysate, we used the spectrophotometric method based on the oxidation reaction of glutathione with 5.5-dithiobis-6.2-nitrobenzoic acid, according to Beutler. In 50 μL of lysate RBCs, we added 200 μL 0.1% ethylenediaminetetraacetic and 385 μL precipitated buffer. After 15 min incubation on ice and 10 min centrifugation on 4000 rpm, an extract was obtained. In 300 μL of the extract, we added 750 μL of sodium phosphate dibasic and 100 μL 5.5-dithiobis-6.2-nitrobenzoic acid. Next, incubated for 10 min and measured in duplicate on 412 nm wavelength. For blind test, instead of the sample, we used distilled water. The calibration curve was constructed to determine the concentrations of the GSH activity in the samples. It was created by four standards with a known amount of glutathione.

Mentioned parameters were determined using Shimadzu UV 1800 spectrophotometer (Shimadzu corporation, Nishinokyo-Kuwabaracho, Nakagyo-KU, Kyoto, Japan) as previously described [12,48].

### 4.7. Cardiac Function Monitored In Vivo

Transthoracic echocardiography evaluation was performed in the last week of chronic protocol, after three-week administration of DATS, using a Hewlett-Packard Sonas 5500, Andover, MA, USA sector scanner equipped with a 15.0 MHz phased-array transducer. Rats were anaesthetized with an intraperitoneal injection of a combination of 0.025 mL ketamine (100 mg/mL; Ketalar, Pfizer Pharmaceuticals, Groton, CT, USA) and 0.025 mL xylazine (20 mg/mL; Xyla, Interchemie, Holland). The cursor was positioned perpendicularly, and images were acquired from M-mode. The following structural variables were measured: interventricular septal wall thickness at end-diastole (IVSd) and end-systole (IVSs), left ventricle (LV) internal dimension at end-diastole (LVIDd) and end-systole (LVIDs), LV posterior wall thickness at end-diastole (LVPWd) and ended-systoleVPWs), as well as fractional shortening (FS) percentage. Average values were obtained from no less than five cardiac sets on M-mode tracking. Additionally, Teicholz formula was used for calculation of ejection fraction (EF) values [52]. Furthermore, echocardiographic measurements were performed in all animals before any manipulation before initiation of DATS, STZ injection, or HFd feeding) to ensure that the cardiodynamic parameters of the animals included in the study were within the physiological range.

Blood pressure (BP) and heart rate (HR) were measured a day before sacrificing animals, by a tail-cuff non-invasive technique (Rat Tail Cuff Method Blood Pressure Systems (MRBP-R), IITC Life Science Inc, Los Angeles, CA, USA) [45]. To eliminate potential variations in blood pressure values due to stress all rats were placed for 30 min in a chamber two days before final BP testing. In each round 8–10 determinations are made, based on which mean values are calculated.

### 4.8. Ex Vivo Experimental Protocol

To examine the heart function in detail, we used the Langendorff apparatus (Experimetria Ltd., Budapest, Hungary) for retrograde heart perfusion and ex vivo-induced I/R injury. Namely, the rats were anaesthetized with an intraperitoneal injection of 0.025 mL ketamine (100 mg/mL; Ketalar, Pfizer, New York, NY, USA) and 0.025 mL xylazine (20 mg/mL; Xyla, Interchemie, Holland) and heart was isolated and perfused with Krebs–Henseleit solution through an aortic cannula on Langendorff apparatus. The left atrium was incised, and mitral valves were separated to provide the placement of the sensor in the LV. After the heart reached a stable steady-state (S), 30 min global ischemia was caused by complete cessation of flow followed by 60 min reperfusion (flow was turned on) [12].

#### 4.8.1. Cardiodynamic and Oxidative Stress Parameters

At the steady-state, as well in the 1st, 3rd, 5th, 10th, 15th, 30th, 45th, and 60th minute of reperfusion cardiodynamic parameters: maximum rate of left ventricular pressure development (marked as dp/dt max, unit: mmHg), a minimum rate of left ventricular pressure development (marked as dp/dt min, unit: mmHg), systolic left ventricular pressure (marked as SLVP, unit: mmHg), diastolic left ventricular pressure (marked as DLVP, unit: mmHg), heart rate (marked as HR, unit: bpm) were monitored. Furthermore, coronary flow (marked as CF unit: mL/min) was followed and coronary venous effluent was collected. All oxidative stress parameters measured in plasma TBARS, NO_2_^−^, O_2_^−^ and H_2_O_2_, were also measured in the coronary venous effluent. Based on these parameters in the coronary venous effluent during I/R injury is directly estimated oxidative stress in the endocardium of the left ventricle and endothelium of coronary circulation. The principle of determination is similar to those in plasma; only a few steps differ [12,48].

#### 4.8.2. Pathohistological Analysis of Myocardial Tissue

After ex vivo protocol on Langendorff apparatus, all hearts were fixed in 4% buffered paraformaldehyde solution on room temperature. Tissues were two times dehydrated with 95% ethanol for 30 min, soaked in xylene for 60 min 60–70 °C and put 12 h in paraffin. The stained tissues were cut into 4 μm sections and then stained with hematoxylin/eosin. To visualize collagen deposition, paraffin heart sections were stained with Picrosirius red (Sigma, Direct Red 80, St. Louis, MO, USA) as previously described [53]. Quantification of red-stained collagen in heart sections stained with Picrosirius red was performed using ImageJ software (National Institute of Health, Bethesda, MD, USA), on 10 fields/section, as previously described [54].

**Figure 10:**
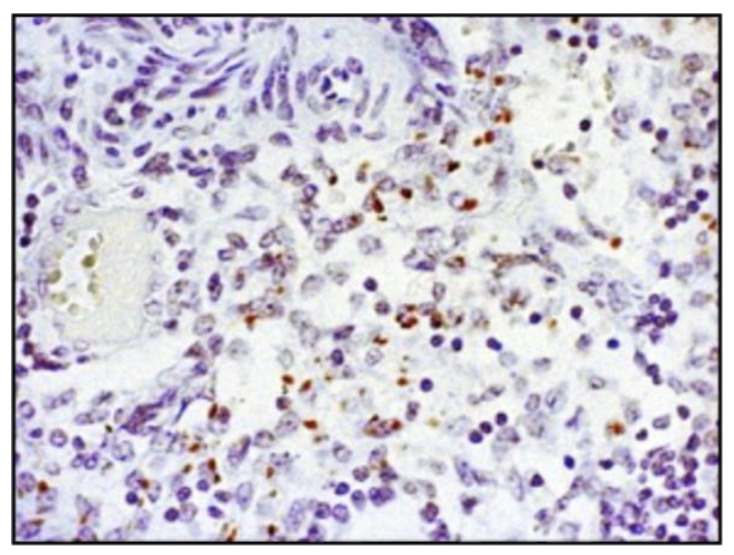
Representative lymph node section (positive external control). Original magnification 40×.

For the violation of the specificity and quality of deoxynucleotidyl transfer-mediated dUTP nick end-labelling (TUNEL) staining in this study [55], we used a positive control. Lymph node tissue, which was previously found to have cells with a TUNEL positive nucleus, was used for this purpose (Figure 10). Positive control sections were treated in the same manner and at the same time as the tested tissues of the heart.

For immunohistochemical staining 5 µm thick heart tissue sections, were dewaxed, rehydrated and treated with citrate buffer (pH 6.0) in the microwave for antigen retrieval. Staining was visualized by using the EXPOSE Rabbit specific HRP/DAB detection IHC Kit (ab80437, Abcam, UK) and sections were counterstained with Mayer’s hematoxylin. The slices were incubated with cardiac troponin T (ab209813), vimentin (working dilution 1:100; Novocastra, Burlingame, CA, USA), recombinant anti-Bax (ab32503), Bcl2 (ab32124), anti-cleaved Caspase 3 (ab2302) and anti-HSP70 (ab5439) overnight at room temperature. Sections were photomicrographed with a digital camera mounted on a light microscope (Olympus Corporation, BX51, Shinjuku-ku, Tokyo, Japan), digitized and analyzed. The analysis was performed on 10 fields/section (×40) using ImageJ software (National Institute of Health, Bethesda, MD, USA). Results are presented as mean count of positively stained cells per field or percentage of immunoreactive area.

Microscopic analysis of the hearts longitudinal sections of all rats was performed by a pathology specialist. To avoid bias and maximizing objectivity, the pathologist was blinded to the sample group. The emphasis was on histologic changes of the left ventricle, and primarily on the lateral wall, septum, and apex.

#### 4.8.3. Expression of Antioxidative, (anti)Apoptotic and (anti)Inflammation Genes in the LV of Heart Tissue

The total RNA (μg) from LV tissue samples was isolated using TRIzol reagent (Invitrogen, Carlsbad, CA, USA) according to the manufacturer’s protocol. Total RNA (μg) was reverse transcribed using High Capacity cDNA Reverse Transcription Kit (Applied Biosystems, Foster City, CA, USA). Real-time quantitative polymerase chain reaction (RT-PCR) was performed using Thermo Scientific Luminaris Color HiGreen qPCR Master Mix (Applied Biosystems, Foster City, CA, USA) and mRNA specific primer for oxidative stress markers: SOD-1, SOD-2, eNOS; (anti)apoptotic markers: Bax, Bcl-2, caspase-3, caspase-9; (anti)inflammation markers: NFkB, TNFα, IL-6, IL-10, and IL-17A.

β-actin (Invitrogen, Carlsbad, CA, USA) was used as a housekeeping gene (Table 1). PCR reactions were performed in a Mastercycler ep realplex (Eppendorf, Hamburg, Germany). Data were analyzed and relative gene expression was calculated according to Livak and Schmittgen.

### 4.9. Statistical Analysis

All presented data are shown as mean ± standard deviation (SD). Figures and tables were made in Microsoft Excel for Mac version 16. Unless otherwise stated, data were assessed by one-way ANOVA followed by Tukey’s multiple comparison post hoc test, using IBM-SPSS, version 20. *p* values less < 0.05 were considered statistically significant.

## 5. Conclusions

The main advantage of this study is the use of DATS that naturally exists in garlic and is unlikely to cause serious side effects, so it can be a great option for clinical relevance. It is important to note that the efficacy of DATS is impossible to achieve just by eating garlic, but further clinical studies in which the optimized formulations of DATS would be applied are needed. In our experimental study, it seems to be well-tolerated, but since its structure is sensitive to chemical transformations, poor water solubility, and the generation of various by-products after H_2_S release [56], larger multi-faceted studies are necessary for a full DATS pharmacodynamic and pharmacokinetic profile. Although it should be kept in mind that not all findings obtained on animals can be directly translated into humans [57], the results of our study are an important cornerstone for future clinical studies in which various formulations of DATS would be examined.

Furthermore, we previously described the positive effects on the cardiovascular system in rats with chemically induced diabetes mellitus type 1; however, the effects of DATS on rats with MetS is barely known. Although we accept the fact that mechanisms through which DATS accomplished its protective effects are similar, used model by itself is completely pathophysiological different. Taken together our results undoubtedly show protective effects of DATS and its potential to control metabolic disorders and prevent their cardiovascular complications. In addition to the previous report which demonstrates antiapoptotic, antioxidative and anti-inflammatory effects of DATS, in this study we also identified its potential to alter levels of homocysteine, H_2_S, lipid status, blood pressure and fibrosis. The relationship between DATS and HSP70 is barely known and with this study, we show that it is possible for DATS to achieve cardioprotection by triggering high expression of HSP70. In this way, we attained a deeper insight into the mechanisms of the protective effects of DATS.

Based on our findings, DATS, a phytochemical derived from garlic, has the potential to be used as a herbal-based supplement in the management of MetS and its related manifestations. The mechanism underlying the cardioprotective effects of DATS against ex vivo induced I/R injury in MetS hearts could be mainly attributed to the ability of this compound to ameliorate oxidative stress, apoptosis and inflammation in the heart. These results suggest that diallyl trisulfide is at least partially responsible for the epidemiologically established cardioprotective and anti/MetS effect of garlic.

## Figures and Tables

**Table 1:** Primers used for RT-PCR analysis.

	Left Primers	Right Primers
β-actin	GATCAGCAAGCAGGAGTACGAT	GTAACAGTCCGCCTAGAAGCAT
SOD-1	TGAAGAGAGGCATGTTGGAGAC	CACACGATCTTCAATGGACACA
SOD-2	AATCAACAGACCCAAGCTAGGC	CACAATGTCACTCCTCTCCGAA
eNOS	GAGGGAGTCAGCCTAAATCCTG	ATCAAAGCATACGAAGAGGGCA
Bcl-2	GCAAAGCACATCCAATAAAAGCG	GTACTTCATCACGATCTCCCGG
Bax	GCTACAGGGTTTCATCCAGGAT	ATGTTGTTGTCCAGTTCATCGC
Caspase-3	GGAAGATCACAGCAAAAGGAGC	GCAGTAGTCGCCTCTGAAGAAA
Caspase-9	TGTACTCCAGGGAAGATCGAGA	CGTTGTTGATGATGAGGCAGTG
NF-kB	GTTTGGTTTGAGACATCCCTGC	CTGTCTTATGGCTGAGGTCTGG
TNF-α	GAAAGCATGATCCGAGATGTGG	CAGGAATGAGAAGAGGCTGAGG
IL-6	GATACCACCCACAACAGACCAG	GTGCATCATCGCTGTTCATACA
IL-10	CTTACTGGCTGGAGTGAAGACC	CTGGGAAGTGGGTGCAGTTATT
IL-17	GCAAGAGATCCTGGTCCTGAAG	AGGTCTCTGTTTAGGACGCATG
